# Deficits in LTP Induction by 5-HT2A Receptor Antagonist in a Mouse Model for Fragile X Syndrome

**DOI:** 10.1371/journal.pone.0048741

**Published:** 2012-10-31

**Authors:** Zhao-hui Xu, Qi Yang, Lan Ma, Shui-bing Liu, Guang-sheng Chen, Yu-mei Wu, Xiao-qiang Li, Gang Liu, Ming-gao Zhao

**Affiliations:** 1 Department of Pharmacology, School of Pharmacy, Fourth Military Medical University, Xi'an, China; 2 Department of Pathology, Xi'an Child's Hospital, Xi'an, China; 3 Department of Orthopaedics and Traumatology, Nanjing General Hospital of Najing Military Commend, PLA, Najing, China; University of Victoria, Canada

## Abstract

Fragile X syndrome is a common inherited form of mental retardation caused by the lack of fragile X mental retardation protein (FMRP) because of *Fmr1* gene silencing. Serotonin (5-HT) is significantly increased in the null mutants of Drosophila *Fmr1*, and elevated 5-HT brain levels result in cognitive and behavioral deficits in human patients. The serotonin type 2A receptor (5-HT2AR) is highly expressed in the cerebral cortex; it acts on pyramidal cells and GABAergic interneurons to modulate cortical functions. 5-HT2AR and FMRP both regulate synaptic plasticity. Therefore, the lack of FMRP may affect serotoninergic activity. In this study, we determined the involvement of FMRP in the 5-HT modulation of synaptic potentiation with the use of primary cortical neuron culture and brain slice recording. Pharmacological inhibition of 5-HT2AR by R-96544 or ketanserin facilitated long-term potentiation (LTP) in the anterior cingulate cortex (ACC) of WT mice. The prefrontal LTP induction was dependent on the activation of NMDARs and elevation of postsynaptic Ca^2+^ concentrations. By contrast, inhibition of 5-HT2AR could not restore the induction of LTP in the ACC of *Fmr1* knock-out mice. Furthermore, 5-HT2AR inhibition induced AMPA receptor GluR1 subtype surface insertion in the cultured ACC neurons of *Fmr1* WT mice, however, GluR1 surface insertion by inhibition of 5-HT2AR was impaired in the neurons of *Fmr1*KO mice. These findings suggested that FMRP was involved in serotonin receptor signaling and contributed in GluR1 surface expression induced by 5-HT2AR inactivation.

## Introduction

Fragile X syndrome (FXS) is the most common inherited form of mental retardation affecting approximately 1∶4,000 males and 1∶6,000 females. This disease is caused by the lack of fragile X mental retardation protein (FMRP) because of *Fmr1*gene silencing [1], [2]. FMRP is found in dendritic spines besides neuronal soma; it is involved in the biochemical regulation of local-dendritic protein synthesis. The primary symptom of FXS is mental retardation. Previous studies suggest that FMRP acts as a local regulator of synaptic plasticity [1], [3], [4], [5]. In our previous studies, long-term potentiation (LTP) was completely abolished [6] in the anterior cingulate cortex (ACC) of *Fmr1* knock-out (KO) mice; the induction of LTP by D1 receptor activation was also impaired [7]. Despite progress on the determination of the etiology of FXS, the mechanism underlying *Fmr1* mutation that results in a devastating syndrome, including altered neural development, cognitive impairment, childhood epilepsy, and autism, remains unknown [8].

The prefrontal cortex (PFC), including ACC, regulates learning and memory, substance dependence, and pain [9], [10], [11], [12]. It receives numerous inputs from cortical and subcortical areas. Previous reports indicated that dopamine (DA) and serotonin are significantly increased in the null mutants of Drosophila *Fmr1*. Elevated brain levels of DA and serotonin result in cognitive and behavioral deficits in human patients [13]. The serotonin type 2A receptor (5-HT2AR) is highly expressed in the cerebral cortex; it modulates cortical functions via its actions on pyramidal cells and GABAergic interneurons [14], [15], [16]. Inhibition of 5-HT2AR markedly potentiates N-methyl-D-aspartate (NMDA) response and excitatory postsynaptic current (EPSC) in CA1 hippocampus pyramidal cell [17]. These studies suggest a significant association between NMDA and 5-HT2A receptors; they also imply that drugs with potent 5-HT2A antagonistic actions may be beneficial in improving cognition in schizophrenia by normalizing NMDA receptor function [18].

Our previous study identified that FMRP acts as a key messenger for DA modulation in the forebrain; our study also provided insights into the cellular and molecular mechanisms underlying FXS [7]. With 5-HT2AR and FMRP both regulating synaptic plasticity, the lack of FMRP may affect serotoninergic activity. However, the connection between FMRP and serotonin modulation has not been established. In this study, we found that FMRP contributes to the modulation of GluR1 receptor synaptic insertion by 5-HT2AR as well as to the induction of LTP in the ACC.

**Figure 1:**
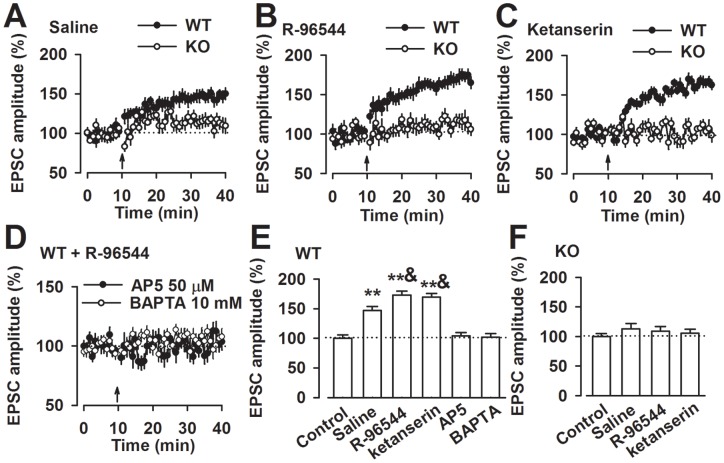
LTP facilitation by R-96544 is impaired in the ACC of *Fmr1* KO mice. (**A**) The pairing training produced a significant, long-lasting potentiation of synaptic responses in WT mice (n = 13 slices/5 mice); LTP was lost in ACC pyramidal neurons of *Fmr1* KO mice (n = 11 slices/6 mice). (**B**) R-96544 paired with training facilitated LTP induction in WT mice (n = 11 slices/5 mice); R-96544 failed to facilitate LTP induction in *Fmr1* KO mice (n = 12 slices/5 mice). (**C**) Ketanserin facilitated LTP induction in *Fmr1* WT mice (n = 12 slices/5 mice); Ketanserin failed to facilitate LTP induction in *Fmr1* KO mice (n = 12 slices/5 mice). (**D**) LTP was blocked in the presence of AP-5 (7 slices/3 mice) and BAPTA (n = 9 slices/4 mice) in the pipette solution in WT mice. (**E**) Summary of the effects of R-96544, ketanserin, AP-5, and BAPTA on LTP in the WT mice. **P<0.01 compared to baseline control; ^&^ *P*<0.05 compared to saline control of LTP. (**F**) Summary of the effects of R-96544 and ketanserin on LTP in the *Fmr1* KO mice.

## Results

### Impaired facilitation of synaptic LTP by R-96544 in *Fmr1* knock-out mice

We examined synaptic potentiation in the ACC, which are structures known to be important in learning and fear memory [11], [19]. LTP was induced by pairing presynaptic stimulation with postsynaptic depolarization (see Materials and Methods). The pairing training produced a significant, long-term potentiation of synaptic responses in wild-type (WT) mice (mean, 147.2%±6.5% of baseline; *n* = 13 slices/5 mice; *P*<0.01 compared with baseline responses before the pairing training) (Figure 1A). However, the pairing training produced a completely inhibited synaptic potentiation in slices of *Fmr1* knock-out (KO) mice (112.7%±9.3%; *n* = 11 slices/6 mice; *P*>0.05 compared with baseline responses) (Figure 1A). To detect the effects of 5-HT2A antagonists on the LTP induction, 5-HT2A antagonists were applied during the baseline recording and paring training, after the training, the drugs were washed out. Bath application of the 5-HT2A receptor antagonist, R-96544 (5 μM) or ketanserin (5 μM), for 30 min significantly enhanced the amplitude of LTP (R-96544: 172.8%±6.9%, n = 11 slices/5 mice; ketanserin: 169.8%±6.3%, n = 12 slices/5 mice; *P*<0.05 versus pairing training only; Figures 1B, C, E, and F). This result suggests that inhibition of 5-HT2AR facilitates LTP induction. However, this facilitation of LTP in WT mice by R-96544 or ketanserin was inhibited in *Fmr1* KO mice (R-96544: 108.7%±8.0%, n = 12 slices/5 mice; ketanserin: 105.8%±6.8%, n = 12 slices/5 mice; *P*>0.05 versus baseline responses Figures 1B, C, E and F). This result demonstrates that serotoninergic modulation of LTP was impaired in *Fmr1* KO mice; therefore, FMRP was required for serotoninergic modulation of LTP in the ACC.

**Figure 2:**
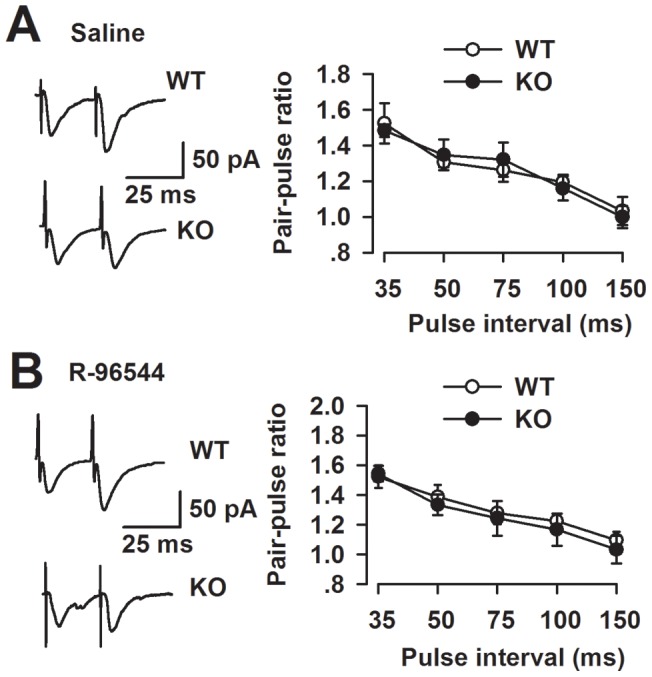
LTP facilitation by R-96544 is through postsynaptic mechanisms. Paired-pulse facilitation in mice before and after perfusion of R-96544 (the ratio of EPSC2/EPSC1) was recorded with intervals of 35, 50, 75, 100, and 150 ms. (**A**) Left panel: representative traces of PPF with an interval of 50 ms recorded in the WT and KO mice ACC. Right panel: PPF was similar at each interval in WT and KO mice in the saline control (n = 9 slices/5 mice in each group). Open circles, from WT slice; filled circles, from KO slice. (**B**) Left panel: representative traces of PPF with intervals of 50 ms recorded i*n the WT and KO* mice. Right panel: PPF was not altered in the WT and KO mice before and after perfusion of R-96544 (n = 10 slices/5 mice in each group). Open circles, from WT slice; filled circles, from KO slice.

We applied a selective NMDAR antagonist, AP5 (50 μM), to determine whether NMDAR activation was required for the prefrontal LTP induction. The results showed that LTP was completely blocked in the presence of AP5 in WT mice (104.2%±6.3%, n = 9 slices/4 mice; Figure 1D and E). Similarly, LTP was abolished by 10 mM 1,2-bis(o-aminophenoxy) ethane-N,N,N′,N′-tetraacetate (BAPTA) in pipette solution (102.6%±6.8%, n = 7 slices/3 mice; Figure 1D and E), indicating that LTP induction depends on the activation of NMDARs and elevation of postsynaptic Ca^2+^ concentrations.

**Figure 3:**
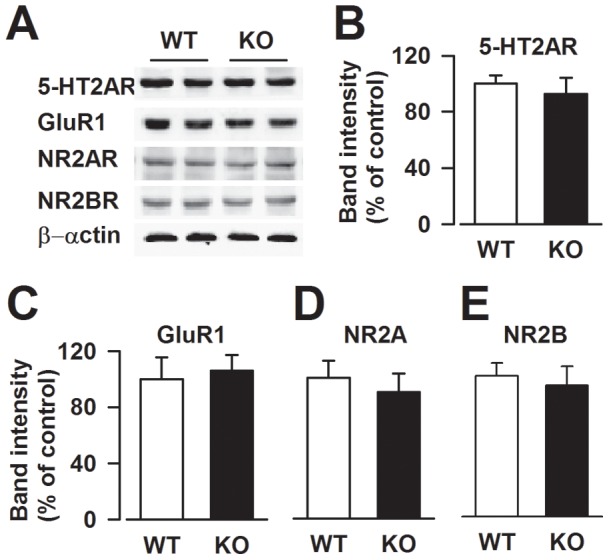
Expression of 5-HT2AR and glutamate receptor subunits. (**A**) Representatives of western blot. There were no differences in 5-HT2AR (**B**), GluR1 (**C**), NR2A (**D**), and NR2B (**E**) expression between the WT and *Fmr1* KO mice ACC. n = 4 mice per group.

### No changes of presynaptic glutamate release by blockade of 5-HT2A

In the different regions of the hippocampus, both presynaptic and postsynaptic mechanisms contribute to the expression of LTP [20]. To determine whether presynaptic and/or postsynaptic mechanisms are involved in the serotoninergic modulation of LTP, we measured paired-pulse facilitation (PPF) in the ACC. PPF is a phenomenon by which a second synaptic stimulation of equal magnitude evokes a larger synaptic response than the first; PPF has been used as a tool to implicate the presynaptic probability of transmitter release [21], [22]. Figure 2 shows that no significant difference was observed in PPF induction at five different intervals between WT and *Fmr1* KO mice in the presence of R-96544 (5 μM) in the ACC. The data suggest that inhibition of 5-HT2A does not change the presynaptic release during the modulation of LTP induction.

**Figure 4:**
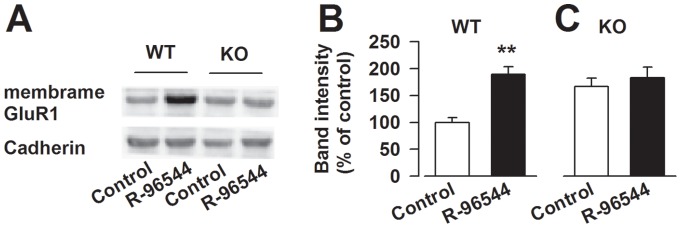
Impaired surface expression of GluR1 by R-96544 in *Fmr1* KO neurons. (A) Representatives of western blot. (B) Surface expression of GluR1 was increased after treatment with 5-HT2AR antagonist R-96544 in the PFC neurons form WT mice (n = 4 dishes). R-96544 failed to trigger GluR1 surface trafficking in the neurons from *Fmr1* KO PFC neurons (n = 4 dishes). **P<0.01 compared to control.

### Expression of 5-HT2A and glutamate receptors in the ACC

To further explore the synaptic mechanisms behind the impairment of serotoninergic modulation of LTP, we examined 5-HT2A receptor and glutamate receptor subunits expression in the ACC from WT and *Fmr1*KO mice. As shown in Figure 3, no differences in the expression of the 5-HT2A receptor were observed between the WT and *Fmr1* KO mice as well as in the expression of GluR1 and NMDA receptor subunits NR2A and NR2B under basal conditions (Figures 3A–E). Therefore, the impaired serotoninergic modulation of synaptic potentiation was not caused by a defect in the expression of 5-HT2A and glutamate receptors.

### Impaired surface expression of GluR1 stimulated by R-96544 in *Fmr1* KO neurons

FMRP acts downstream from the NMDA receptor and contributes to memory-related synaptic potentiation in the ACC of the PFC [7], [11], [12]. To investigate the role of the 5-HT2A receptor signaling pathway in synaptic plasticity, we tested the effects of R-96544 on AMPA receptor GluR1 subunits surface trafficking in cultured PFC neurons in the presence of 1 μM serotonin. The surface expression of GluR1 was increased after treatment with 5-HT2AR antagonist R-96544 (5 μM) (*P*<0.01 versus control, n = 4 dishes; Figure 4). This finding demonstrates that inhibition of the 5-HT2A receptor can facilitate the surface expression of AMPA GluR1 receptors. However, the surface expression of GluR1 stimulated by R-96544 was impaired in *Fmr1* KO PFC neurons (*P*>0.05, n = 4 dishes; Figure 4). These results indicate that FMRP is involved in serotonin receptor signaling and contributed to GluR1 surface expression induced by 5-HT2A receptor inactivation.

## Discussion

In this study, we showed that inhibition of the 5-HT2A receptor modulates glutamatergic synaptic transmission and synaptic plasticity in the ACC, facilitates the induction of LTP in the ACC, and increases GluR1 surface expression in PFC neurons. However, all these processes were inhibited in *Fmr1* KO neurons. Therefore, the results suggest that FMRP is required for the serotoninergic modulation of LTP in the ACC.

### Serotonergic modulation of long-term synaptic plasticity

Synapses in the cingulate cortex undergo plasticity; they are involved in higher brain functions such as learning and memory [12]. Previous studies indicated that 5-HT plays key roles in the maintenance and regulation of balance between potentiation and depression of synaptic networks in the hippocampus [23]. The 5-HT2A receptor antagonist markedly potentiates NMDA response and EPSC in CA1 hippocampus pyramidal cell [17]. Alternatively, the 5-HT2A receptor antagonist can prevent impairment in performance by NMDA receptor blockade in rat PFC [24], [25], [26], [27]. This function is regulated through attenuating the suppression of the NMDA receptor blockade-induced release of cortical glutamate [28]. Therefore, a mutual interaction occurs between serotoninergic and glutamatergic neurotransmissions. In the PFC, including the ACC, activation of the NMDA receptor subunits NR2B and NR2A is critical for the induction of long-term cingulate synaptic plasticity [29], [30]. Previous studies found that the 5-HT2A antagonist enhances the induction of long-term potentiation of CA1 synapses [17]. The present study shows that inhibition of the 5-HT2A receptor facilitates the induction of LTP at ACC synapses. This facilitation of LTP was inhibited in the presence of NMDAR antagonist AP-5 and BAPTA, indicating dependence on the activation of NMDARs and elevated postsynaptic Ca^2+^ concentrations.

### FMRP, 5-HT modulation of AMPA receptors, and synaptic potentiation

Our previous study showed that FMRP acts as a key messenger for DA-mediated modulation of excitatory transmission in forebrain neurons [7]. FMRP is required for the surface expression of AMPA GluR1 receptors in response to D1 receptor activation. Facilitation of LTP by D1 receptor activation was consistently absent in the cingulate region of the PFC of *Fmr1* KO mice. Recent studies have focused on the interaction between DA and 5-HT receptors. In the intact striatum, studies have consistently demonstrated that intrinsic 5-HT2 receptors modify DA function; furthermore, the divergent roles of 5-HT2A and 5-HT2C receptor subtypes have been postulated [31], [32]. In the present study, we compared the effects of 5-HT2A receptor inhibition on GluR1 surface trafficking in cultured PFC neurons to illustrate the role of FMRP in serotoninergic modulation of GluR1 receptors. We found that inhibition of the 5-HT2A receptor increases GluR1 surface trafficking in ACC neurons; however, this increase was significantly attenuated in *Fmr1* KO neurons. This is consistent the electrophysiological recording showing the lost of LTP modulation in the KO nice. Further studies are needed to figure out the molecular signaling involving this disorder.

Several studies proved that the 5-HT2A antagonist can facilitate NMDA receptor-mediated transmission [17], [24], [25], [26], [27], [33]. Moreover, 5-HT2A antagonist significantly increased the amplitude and duration of excitatory postsynaptic potentials and currents evoked by electrical stimulation of the forceps minor [33]. We found that 5-HT2A antagonist facilitates cingulate prefrontal LTP through increased synaptic incorporation of GluR1. The unaltered PPF suggests that the 5-HT2A antagonist does not alter the presynaptic release in the ACC. However, 5-HT reportedly enhances synaptic transmission by increasing the release of glutamate via presynaptic 5-HT2A receptors in rat dorsolateral septal nucleus [34]. The difference between the pre and postsynaptic mechanisms of the 5-HT2A receptor can be attributed to the different distributions of 5-HT receptors in different brain regions. Furthermore, we cannot rule out the possible presynaptic modulation of inhibitory transmission by serotoninergic modulation [35], which was blocked in our recordings.

#### Conclusion

This study, together with our previous study, demonstrated 5-HT modulations of synaptic transmission and plasticity at the ACC. GluR1 surface expression and LTP facilitation in response to 5-HT2AR inhibition were impaired in the ACC of *Fmr1* KO mice. These findings indicate that FMRP plays a key role in serotoninergic modulation in the forebrain and may provide insights into the cellular and molecular mechanisms underlying FXS.

## Methods

### Animals

For adult brain slice recordings, male mice were 6–8 weeks of age. *Fmr1* knock-out mice (strain name: FVB.129P2-*Fmr1* ^tm1Cgr^/J; stock #:4624) and control wild-type mice on the same strain background (stock # 4828) were purchased from The Jackson Laboratory (Genetics research, Bar Harbor, Maine, USA) and bred in the animal facility of the Fourth Military Medical University. All mice were housed in plastic boxes in groups of 4 under a 12∶12 light cycle with food and water provided *ad libitum*. This study was performed in adherence with the National Institutes of Health guidelines for the use of experimental animals. The Institutional Ethical Committee of the Fourth Military Medical University specifically approved this study.

### Slice preparation

Coronal brain slices (300 μM) from 6–8-week old *Fmr1* WT and KO mice, containing the ACC, were prepared using standard methods [6]. Slices were transferred to submerged recovery chamber with oxygenated (95% O_2_ and 5% CO_2_) artificial cerebrospinal fluid (ACSF) containing (in mM: 124 NaCl, 4.4 KCl, 2 CaCl_2_, 1 MgSO_4_, 25 NaHCO_3_, 1 NaH_2_PO_4_, 10 glucose) at room temperature for at least 1 h.

### Whole-cell recordings

Experiments were performed in a recording chamber on the stage of an Axioskop 2FS microscope with infrared DIC optics for visualization of whole-cell patch clamp recording. Patch-clamp recordings were performed using Axon 200B amplifiers (Axon Instruments, Union City, CA, USA). In the ACC slices, since layer II-III pyramidal neurons are reported as the major location of intra-cortical horizontal pathways [36] and the superficial layers of the ACC receive substantial glutamate input [37], we recorded EPSCs from neurons in layer II-III induced by the stimulation of layer V. AMPA receptor-mediated EPSCs were induced by repetitive stimulations at 0.02 Hz and neurons were voltage clamped at −70 mV. After obtaining stable EPSCs for at least 10 min, LTP was induced by 80 pulses at 2 Hz paired with postsynaptic depolarization at +30 mV. The recording pipettes (3–5 MΩ were filled with solution containing (mM) 145 K-gluconate, 5 NaCl, 1 MgCl_2_, 0.2 EGTA, 10 HEPES, 2 Mg-ATP, and 0.1 Na_3_-GTP (adjusted to pH 7.2 with KOH). Picrotoxin (100 μM) was always present to block GABA_A_ receptor-mediated inhibitory synaptic currents. Access resistance was 15–30 MΩ and monitored throughout the experiment. Recordings were digitized (10 kHz) on-line using a Digidata 1322 (Axon Instruments) and analyzed off-line with Clamfit 9.0 (Axon Instruments). Data were discarded if access resistance changed more than 15% during an experiment.

### Primary culture of prefrontal cortical neurons

The experiments were performed on C57 mice (embryonic, 18 days old of both genders). The Animal Care and Use Committee of the Fourth Military Medical University approved all of the animal protocols used. Cultured prefrontal cortex neurons were prepared as previously described [7]. Briefly, the prefrontal cortex was dissected, minced, and trypsinized for 15 min using 0.125% trypsin (Invitrogen, Carlsbad, USA). The cells were seeded onto 100 mm dishes precoated with 50 μg/ml poly-d-lysine (Sigma) in water and grown in Neurobasal-A medium (Invitrogen) supplemented with B27 and 2 mM GlutaMax (Invitrogen). In the B27/Neurobasal medium, glial growth was reduced to less than 0.5% of the nearly pure neuronal population, as assessed using immunocytochemistry for glial fibrillary acidic protein and neuron specific enolase [38]. The cultures were incubated at 37°C in 95% air/5% carbon dioxide with 95% humidity. The cultures were used for experiments on the 10th day *in vitro* (DIV 10). No serotonin was added in the experiment; only 5-HT2AR antagonist R-96544 (5 μM, TOCRIS, Bristol, UK) was added in the medium for 6 h before the cells harvested.

### Membrane protein preparation

Surface protein samples were separated by superspeed centrifuge. Briefly, cultures were washed three times with ice-cold PBS. Cells were collected and homogenated in buffer A containing 0.32 M sucrose, 5 mM Tris-HCl (PH 7.5), 120 mM KCl, 1 mM EDTA, 0.2 mM PMSF, 1 μg/ml Leupeptin, 1 μg/ml Pepstatin A and 1 μg/ml Aprotinin. The lysate was centrifuged at 100,000 g for 1 h at 4°C. Carefully remove and discard the supernatant, the precipitation was obtained and suspended with appropriate buffer B containing 20 mM HEPES (PH 7.5), 10% glycerol, 2% Triton X-100, 1 mM EDTA, 0.2 mM PMSF, 1 μg/ml Leupeptin, 1 μg/ml Pepstatin A and 1 μg/ml Aprotinin. After incubating on ice for 2 h, the suspension was centrifuged at 10,000 g for 30 min at 4°C. The majority of membrane protein was in the supernatant.

### Western blot

Western blot analysis was performed as described previously [7]. Equal amounts of protein (50 μg) from the cultures were separated and electrotransferred onto PDVF membranes (Invitrogen), which were probed with antibodies for 5-HT2AR (dilution ratio 1∶200, Abcam, Cambridge, MA, #ab66049), GluR1 (dilution ratio 1∶300, Millipore, Billerica, MA, #AB1504), NR2A (dilution ratio 1∶200, Millipore, Billerica, MA, #MAB5216), NR2B (dilution ratio 1∶1000, Millipore, Billerica, MA, #MAB5780), and with β-actin (dilution ratio 1∶10000, Sigma, MO, #A5316) as loading control. For data quantitation, band intensity was expressed relative to the loading control (β-actin). The membranes were incubated with horseradish peroxidase conjugated secondary antibodies (anti-rabbit IgG for the primary antibodies), and bands were visualized using an ECL system (Perkin Elmer).

### Data analysis

Results were expressed as mean ± SEM. Statistical comparisons were performed using one- or two-way analysis of variance (ANOVA) using the Student-Newmann-Keuls for post-hoc comparisons. In all cases, *P*<0.05 was considered statistically significant.
